# Chemerin in Pulmonary Fibrosis: Advances in Mechanistic and Fundamental Research

**DOI:** 10.3390/biom15101469

**Published:** 2025-10-17

**Authors:** Yongshuai Jiang, Ziyang Li, Zhenghang Huang, Junsheng Dong, Li Qian

**Affiliations:** 1 Guangling College, Yangzhou University, Yangzhou 225009, China; ysjiang0225@yzu.edu.cn; 2 School of Basic Medical Sciences & School of Public Health, Faculty of Medicine, Yangzhou University, Yangzhou 225009, China; yzucris@gmail.com (Z.L.); zhenghanghuang32@gmail.com (Z.H.); 3 Key Laboratory of the Jiangsu Higher Education Institutions for Integrated Traditional Chinese and Western Medicine in Senile Diseases Control, Yangzhou University, Yangzhou 225009, China; 4 College of Veterinary Medicine, Yangzhou University, Yangzhou 225009, China

**Keywords:** chemerin, chemerin receptor, lung fibrosis, inflammation, molecular target

## Abstract

Pulmonary fibrosis is a progressive interstitial lung disease that involves stimulated growth of fibroblasts, over-deposition of extracellular matrix (ECM), and permanent damage of the lung structure. Among its various forms, idiopathic pulmonary fibrosis (IPF) is the most common and life-threatening type with few treatment options and a poor prognosis. Such obstacles highlight the urgency to find new molecular targets by better understanding the cellular and signaling processes that contribute to the pathogenesis of the disease. Chemerin is an adipokine and chemoattractant protein that has recently come into the limelight as a major controller of immune cell trafficking, inflammation, and tissue remodeling. Its biological activity is mainly mediated by binding to its receptors Chemokine-like receptor 1 (CMKLR1), G protein-coupled receptor 1 (GPR1), and C-C chemokine receptor-like 2 (CCRL2), and has been linked to numerous pathological conditions, such as metabolic diseases, cancer, and inflammatory diseases. Emerging data now indicate that chemerin can also be a key factor in the initiation and progression of pulmonary fibrosis. The aim of the review is to overview the existing evidence regarding regulatory processes of chemerin expression, signaling pathways, and effects of this protein in cells in the fibrotic lung microenvironment. Moreover, we will comment on the findings of in vitro and in vivo experiments supporting the possibility of chemerin as a promising molecular target in basic research on pulmonary fibrosis.

## 1. Introduction

Chemerin is encoded by the *RARRES2* gene. It is released as an inactive precursor and requires proteolytic cleavage at its C-terminus to achieve biologic activity. Chemokine-like receptor 1 (CMKLR1), G protein-coupled receptor (GPCR), is the main receptor of the bioactive form of chemerin and plays a role in its physiological actions [1,2]. Chemerin mediates diverse functions upon binding, including the recruitment of immune cells such as macrophages, dendritic cells, and natural killer (NK) cells, as well as enhances glucose uptake and promotes adipogenesis by upregulating adipocyte differentiation pathways [3].

The liver and white adipose tissue are the two main sites of chemerin synthesis, although epithelial cells, endothelial cells, and alveolar macrophages can also synthesize chemerin locally in the lung [4]. Pro-inflammatory cytokines like tumor necrosis factor-alpha (TNF-α) and interleukin-1 beta (IL-1β), oxidative stress and hypoxia, which are typically upregulated in the fibrotic lung microenvironment, tightly regulate chemerin expression and secretion [5]. It has been proposed that chemerin is involved in local immune responses and tissue repair, as evidenced by the dynamic regulation of chemerin levels in response to tissue injury and inflammation.

The main receptor mediating the biological action of chemerin is CMKLR1, but GPR1 and CCRL2 can play a role in some physiological or pathological conditions [6]. Activation of CMKLR1 leads to the activation of intracellular signaling cascades, such as the Phosphatidylionsitol 3-kinase/Protein kinase B(PI3K/Akt), Mitogen-Activated Protein Kinase/Extracellular Signal-Regulated Kinase(MAPK/ERK), and Nuclear Factor kappa-light-chain-enhancer of activated B cells(NF-κB) pathways [7]. Some of the cellular responses regulated by these pathways include cell proliferation, chemotaxis of immune cells, cell proliferation, and survival. These signaling networks could be dysregulated to stimulate chronic inflammation, fibroblast activation, and epithelial cell injury during pulmonary fibrosis, fueling disease progression [8,9].

**Figure 1:**
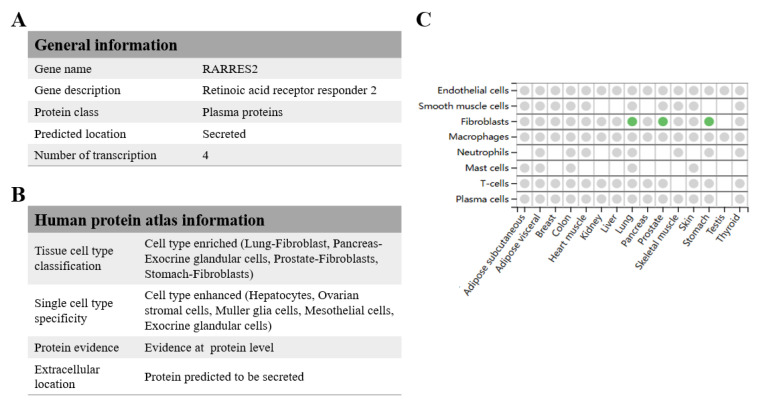
Chemerin is closely associated with pulmonary fibrosis. (**A**) General information of chemerin. (**B**) Human protein atlas information of chemerin. (**C**) The correlation between chemerin and pulmonary fibrosis. The green dot indicates confirmed chemerin protein expression in mesenchymal cells. All these observations are from human tissue cell type (https://www.proteinatlas.org/) (accessed on 6 January 2025) [11,12].

Studies have demonstrated that chemerin contributes greatly to the pathogenesis of this disease, as it interferes with many cellular processes, including inflammation, fibrosis, and tissue remodeling [10]. High concentrations of chemerin have been reported in patients with pulmonary fibrosis, implying that it could be used as a biomarker or therapeutic target in the management of the disease (Figure 1). The discussion of how chemerin promotes fibrosis can help develop new treatment options to reduce lung injury and enhance patient outcomes.

## 2. Elevated Chemerin May Promote Pulmonary Fibrosis

Pulmonary fibrosis is pathologically characterized by the disruption of alveolar architecture, excessive proliferation of fibroblasts and myofibroblasts, collagen accumulation, and thickening of the alveolar septa. These structural alterations impair gas exchange, ultimately leading to progressive respiratory failure. The fibrotic process is primarily driven by chronic alveolar epithelial injury, dysregulated wound healing, and persistent fibroblast activation—often occurring without a clearly identifiable initiating factor, particularly in idiopathic pulmonary fibrosis (IPF) [13]. Fibrotic lesions are typically patchy yet irreversible, contributing to a steady and relentless decline in lung function. Pulmonary fibrosis can be broadly classified into idiopathic and secondary forms. IPF, the most aggressive and lethal subtype, is a diagnosis of exclusion and lacks a known cause. In contrast, secondary pulmonary fibrosis results from identifiable etiologies such as autoimmune diseases, occupational or environmental exposures, infections, and drug-induced toxicity [14]. While each form may involve distinct pathogenic mechanisms, they often share overlapping pathological and molecular features.

**Figure 2:**
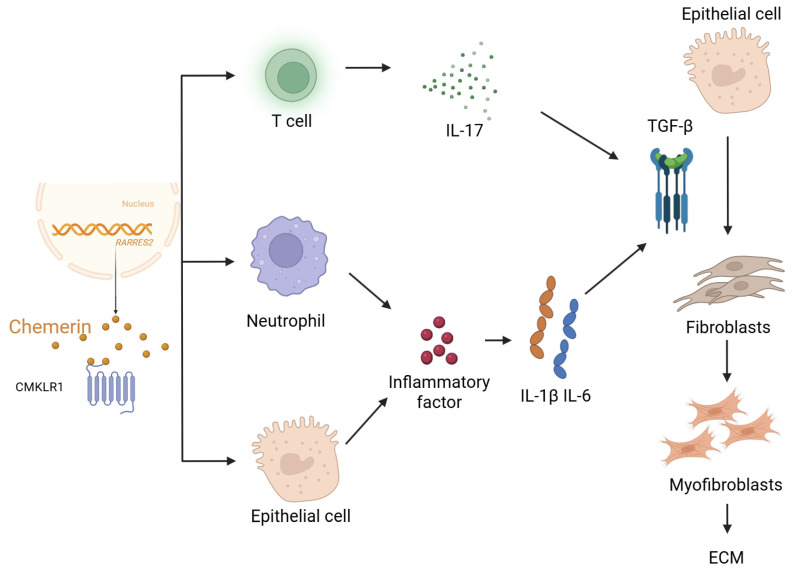
Multiple mechanisms of chemerin in pulmonary fibrosis. Chemerin, encoded by the *RARRES2* gene, is secreted as a precursor and becomes biologically active after proteolytic cleavage. Active chemerin binds to its receptor CMKLR1 on immune and structural cells, initiating downstream responses. Chemerin promotes recruitment and activation of T cells, driving IL-17 production, and of neutrophils, which release inflammatory mediators. Activated epithelial cells further amplify inflammation by secreting cytokines such as IL-1β and IL-6, which in turn stimulate fibroblasts. Together with TGF-β signaling from epithelial cells, these cytokines promote fibroblast activation, proliferation, and differentiation into myofibroblasts, leading to excessive extracellular matrix (ECM) deposition and progressive fibrotic remodeling.

Multiple overlapping mechanisms drive the pathogenesis and progression of pulmonary fibrosis. Chronic inflammation triggered by persistent exposure to irritants or autoimmunity leads to the secretion of pro-fibrotic cytokines such as transforming growth factor-beta (TGF-β). Oxidative stress further exacerbates tissue damage and promotes epithelial–mesenchymal transition (EMT), a process in which alveolar epithelial cells acquire mesenchymal characteristics and contribute to the fibroblast pool. Activated fibroblasts and myofibroblasts serve as principal effectors of fibrosis, producing large amounts of extracellular matrix proteins, especially collagen, which disrupts the normal lung architecture [15,16] (Figure 2). Elucidating these mechanisms is essential for the identification of novel therapeutic targets. Chemerin is an important chemokine that participates in various physiological and pathological processes, including inflammation and immune responses. Recent studies indicate that chemerin may play a significant role in the development of pulmonary fibrosis. Pulmonary fibrosis is characterized by scarring of lung tissue and is commonly seen in conditions such as idiopathic pulmonary fibrosis, systemic sclerosis, and other lung diseases. Chemerin can regulate the activity of macrophages and other immune cells by binding to its receptors, thereby influencing the inflammatory response and the fibrotic process.

It has been discovered that in patients with pulmonary fibrosis, chemerin levels can be increased, and these levels are correlated with the degree of fibrosis. Chemerin might encourage fibroblast proliferation and collagen deposition, thereby accelerating the process of pulmonary fibrosis. Thus, the identification of the chemerin or its signaling pathways as targets can offer new approaches to pulmonary fibrosis treatment. To conclude, chemerin has been implicated in pulmonary fibrosis, and its role in the disease should be investigated further to find new ways of treating similar diseases.

## 3. Functional Role of Chemerin in Pulmonary Fibrosis

### 3.1. Mechanistic Basis for Chemerin’s Involvement in Pulmonary Fibrosis

Chemerin is a chemoattractant protein that has been suggested to play a role in lung fibrosis pathophysiology, with potential widespread roles in the pathogenesis and pathophysiology [17].

In bleomycin-induced pulmonary fibrosis in mice, chemerin and its receptor, CMKLR1, are markedly upregulated in lung tissue [18,19,20], largely under the influence of oxidative stress and pro-inflammatory cytokines characteristic of fibrosis [21]. Although chemerin is primarily synthesized in the liver and white adipose tissue, it can also be synthesized locally within the lungs by epithelial cells, endothelial cells, and alveolar macrophages, further contributing to its local effects in the lung [22,23].

Chemerin exerts its biological effects primarily through CMKLR1, leading to activation of PI3K/Akt, MAPK/ERK, and NF-κB, which collectively drive canonical profibrotic signaling pathways [24,25]. These pathways are closely associated with fibrotic activation, including fibroblast activation, extracellular matrix (ECM) deposition, and modulation of immune responses [26,27]. Notably, chemerin promotes fibroblast proliferation and migration, likely mediated through sustained activation of the Akt pathway [28]. Given this complexity, chemerin’s role may extend beyond pro-inflammatory recruitment. As a potent chemoattractant, chemerin recruits macrophages, dendritic cells, and natural killer cells to sites of epithelial injury [29,30], thereby initiating or exacerbating local inflammation [31]. Moreover, chemerin enhances macrophage secretion of pro-fibrotic cytokines such as TGF-β and IL-6, further accelerating fibrogenesis [32,33].

Historically, chronic inflammation was considered a central driver of pulmonary fibrosis. However, idiopathic pulmonary fibrosis (IPF) is now recognized as a disease with minimal inflammatory infiltrate but extensive epithelial injury, fibroblast activation, and aberrant tissue remodeling [34]. In contrast, hypersensitivity pneumonitis and NSIP are highly inflammatory but less fibrotic, underscoring the heterogeneity of interstitial lung diseases. Meta-analyses of clinical trials have shown that broad immunosuppression is harmful in IPF, overturning the “inflammation causes fibrosis” paradigm [35]. Moreover, some animal models suggest that certain inflammatory responses may be protective, limiting fibrosis progression [36].

Fibroblast activation is central to the fibrotic process. Chemerin has been shown to directly stimulate pulmonary fibroblast proliferation and upregulate the expression of type I and III collagen, thereby promoting ECM accumulation [2]. This may occur via the chemerin–CMKLR1 axis through modulation of TGF-β signaling [30]. Epithelial–mesenchymal transition (EMT) is a key mechanism whereby epithelial cells acquire mesenchymal characteristics during fibrosis [13]. Chemerin facilitates EMT by inducing the expression of transcription factors such as Snail and Twist, downregulating E-cadherin, and upregulating N-cadherin and vimentin [37]. It may also act synergistically with TGF-β1 to stabilize the mesenchymal phenotype [30,38].

Chemerin is secreted as an inactive prochemerin and requires proteolytic cleavage at its C-terminus by serine proteases such as neutrophil elastase, cathepsin G, and plasmin to become biologically active [39]. However, the specific protease network responsible for chemerin activation during fibrogenesis is not fully defined, and its temporal regulation remains unclear. Genetically engineered *RARRES2*^−/−^ (chemerin-deficient) and CMKLR1^−/−^ mice have been used to study metabolic and inflammatory diseases [40], but there is a paucity of data examining their response to bleomycin- or silica-induced lung fibrosis. Similarly, no published studies have yet evaluated whether administration of recombinant active chemerin alone is sufficient to induce pulmonary fibrosis. Addressing these knowledge gaps—through loss-of-function and gain-of-function studies—will be crucial to clarify whether chemerin is a causal driver of fibrosis or a bystander marker of injury [41].

### 3.2. Etiological and Risk Factors Associated with Chemerin in Pulmonary Fibrosis

Exposure to environmental toxins such as silica, asbestos, and cigarette smoke significantly increases Chemerin expression, thereby promoting pulmonary inflammation and fibrosis [13]. Additionally, genetic variants—particularly polymorphisms in the *RARRES2* gene—may modulate individual susceptibility to chemerin-mediated signaling and fibrotic risk [21].

Chemerin expression varies among different forms of interstitial lung disease (ILD), including idiopathic pulmonary fibrosis (IPF), autoimmune-related fibrosis (e.g., systemic sclerosis-associated ILD), and occupational fibrosis [13]. Clinical studies have consistently reported elevated serum and bronchoalveolar lavage fluid (BALF) chemerin levels in IPF patients, correlating with disease progression. Inhalation of environmental toxins (e.g., cigarette smoke, nitrogen dioxide, particulate matter) stimulates pulmonary immune cells to secrete pro-inflammatory cytokines, thereby inducing chemerin upregulation [42,43,44]. This facilitates immune cell infiltration and fibroblast activation, ultimately exacerbating lung tissue injury and fibrotic remodeling [45]. Certain viruses—including Epstein–Barr virus (EBV) and SARS-CoV-2—are linked to fibrotic lung injury [46]. Viral infection triggers immune activation and upregulates chemerin expression in lung tissue and BALF, promoting immune cell recruitment and enhancing fibroblast activation, thereby contributing to post-viral fibrosis [47]. Chemerin may exhibit dual roles by enhancing antiviral immunity while concurrently promoting chronic inflammation and ECM deposition [48,49].

### 3.3. Clinical Relevance of Chemerin in Pulmonary Fibrosis

Serum chemerin levels correlate with FVC% and DLCO%, highlighting its potential as a biomarker. Paralleling CMKLR1 expression in lung tissues [48]. Its non-invasive detectability and correlation with disease activity highlight chemerin’s potential as a diagnostic biomarker.

Chemerin levels negatively correlate with pulmonary function indices such as forced vital capacity (FVC%) and diffusing capacity for carbon monoxide (DLCO%) [50]. Longitudinal studies indicate that elevated chemerin levels are predictive of rapid disease progression and increased risk of acute exacerbations [51,52]. Dynamic associations have been observed between chemerin levels and lung function metrics during disease progression and treatment [53]. Declining chemerin levels in response to anti-fibrotic therapies have been associated with functional stabilization or improvement, underscoring its utility in treatment monitoring and individualized care [54].

### 3.4. Therapeutic Implications of Targeting Chemerin in Pulmonary Fibrosis

**Figure 3:**
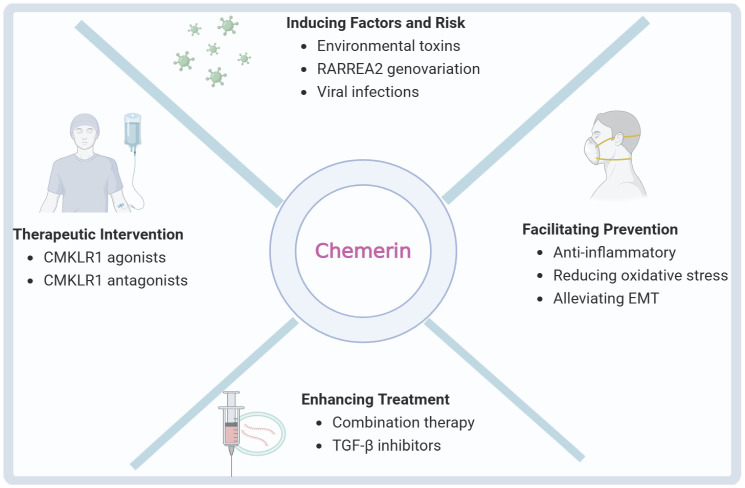
Multiple treatments using chemerin in pulmonary fibrosis.

Several strategies have been proposed to target chemerin signaling in pulmonary fibrosis (Figure 3). These strategies may include Anti-inflammatory agents (e.g., corticosteroids) and anti-fibrotic drugs (e.g., nintedanib, pirfenidone) have been shown to partially downregulate chemerin expression, mitigating inflammatory and fibrotic responses [32,46,55]. Incorporation of chemerin-targeted strategies may augment the efficacy of existing treatments [56].

Beyond these approaches, emerging studies have explored direct modulation of the chemerin–CMKLR1 axis. CMKLR1 agonists such as chemerin-9 have been shown to activate pro-resolving immune pathways and may help restore homeostasis in certain injury contexts [48,57]. Conversely, several groups have reported the discovery of small-molecule CMKLR1 antagonists through high-throughput screening and structure–activity relationship (SAR) optimization [58]. These antagonists competitively block chemerin binding to CMKLR1, thereby preventing downstream PI3K/Akt and NF-κB activation. In preclinical inflammatory models, CMKLR1 antagonists attenuated leukocyte recruitment, reduced cytokine production, and limited tissue damage. Although these compounds have not yet been evaluated in bleomycin- or silica-induced pulmonary fibrosis, they hold promise as orally bioavailable drug candidates with favorable pharmacokinetic properties [59].

Combination strategies may be particularly promising: dual inhibition of chemerin and TGF-β signaling yields enhanced suppression of fibroblast activation and fibrosis progression [1]. Future research should explore whether CMKLR1 antagonists can synergize with antifibrotic therapies such as pirfenidone or nintedanib to improve outcomes. Moreover, chemerin modulation may improve patient tolerance to current treatments by reducing side effects. These approaches are still under investigation, and further research is needed to determine their safety and efficacy in treating pulmonary fibrosis.

## 4. Discussion and Perspectives

Although significant progress has been made in understanding the potential role of chemerin in pulmonary fibrosis, its molecular mechanisms remain incompletely elucidated. Future studies should focus on the following areas:

Current research primarily centers on chemerin-mediated activation of PI3K/Akt, MAPK, and NF-κB signaling pathways via its receptor CMKLR1, affecting fibroblasts and immune cells. However, it remains unclear whether its effects are spatiotemporally dependent across different cell types, including epithelial cells, endothelial cells, and immune cells. Moreover, the potential involvement of chemerin in regulating non-coding RNAs, epigenetic modifications, or cellular metabolism during fibrogenesis warrants further investigation [52,60]. The crosstalk between chemerin and classical profibrotic cytokines such as TGF-β and IL-13 also needs to be clarified [61].

Several studies have reported a significant correlation between chemerin levels in serum and bronchoalveolar lavage fluid (BALF) and disease severity in interstitial lung diseases, including IPF [62]. Future large-scale, multicenter prospective studies are required to evaluate its prognostic value in disease progression, treatment response, and acute exacerbations. Additionally, its potential to complement existing biomarkers, such as KL-6 and SP-D, should be assessed [63].

Preclinical studies have demonstrated that CMKLR1 antagonists or chemerin modulators can alleviate pulmonary fibrosis in animal models [64,65]. However, these findings have yet to be translated into clinical trials. Future drug development should integrate high-throughput screening, structural biology, and pharmacokinetic optimization to enhance the efficacy and safety of chemerin-targeted therapies [66]. Furthermore, the potential synergy between chemerin inhibitors and existing antifibrotic agents, such as pirfenidone and nintedanib, should be explored to enable more precise and individualized treatment approaches [67,68]. The development of selective CMKLR1 antagonists represents an attractive therapeutic strategy. Further studies should test these compounds in combination with standard antifibrotic drugs to determine whether dual inhibition yields additive or synergistic benefit.

Although chemerin knockout (chemerin-KO) mice have been generated and widely used to study immune regulation, metabolic homeostasis, and tumor biology [69,70], there are currently no published studies systematically examining pulmonary fibrosis in chemerin-deficient mice [71,72]. This represents an important gap in our understanding of chemerin biology. Given that chemerin is involved in leukocyte trafficking, fibroblast activation, and tissue remodeling, chemerin-KO models would provide valuable insight into whether the absence of chemerin protects from, exacerbates, or has no effect on fibrosis development. Future studies employing chemerin-KO mice in well-established pulmonary fibrosis models (e.g., bleomycin-induced fibrosis) are therefore warranted to clarify the in vivo contribution of chemerin to fibrotic lung pathogenesis [70,71,72,73,74].

## 5. Conclusions

Chemerin, a multifunctional protein involved in inflammatory regulation, immune cell chemotaxis, and tissue remodeling, plays a pivotal role in the pathogenesis of pulmonary fibrosis. Through its receptor CMKLR1 and downstream signaling pathways, chemerin contributes to fibroblast activation, ECM deposition, immune cell infiltration, and epithelial–mesenchymal transition, forming a key regulatory axis within the fibrotic microenvironment. Clinically, elevated chemerin expression is closely associated with disease activity and holds promise as a diagnostic and prognostic biomarker. In addition, evidence from preclinical models supports its potential as a therapeutic target. Future research should continue to dissect its molecular actions, validate its clinical biomarker potential, and promote the development of chemerin-targeted interventions. These efforts may ultimately contribute to the advancement of early diagnosis and precision therapy in pulmonary fibrosis.

## Data Availability

No data associated in the manuscript.
